# An RNA-Seq Analysis of Grape Plantlets Grown *in vitro* Reveals Different Responses to Blue, Green, Red LED Light, and White Fluorescent Light

**DOI:** 10.3389/fpls.2017.00078

**Published:** 2017-01-31

**Authors:** Chun-Xia Li, Zhi-Gang Xu, Rui-Qi Dong, Sheng-Xin Chang, Lian-Zhen Wang, Muhammad Khalil-Ur-Rehman, Jian-Min Tao

**Affiliations:** ^1^College of Horticulture, Nanjing Agricultural UniversityNanjing, China; ^2^College of Agriculture, Nanjing Agricultural UniversityNanjing, China; ^3^Tropical Crops Genetic Resources Institute, Chinese Academy of Tropical Agricultural SciencesDanzhou, China; ^4^College of Life Science and Food Engineering, Huaiyin Institute of TechnologyHuaian, China

**Keywords:** RNA-seq, grape, differentially expressed genes (DEGs), growth, physiological, light-emitting diodes (LEDs)

## Abstract

Using an RNA sequencing (RNA-seq) approach, we analyzed the differentially expressed genes (DEGs) and physiological behaviors of “Manicure Finger” grape plantlets grown *in vitro* under white, blue, green, and red light. A total of 670, 1601, and 746 DEGs were identified in plants exposed to blue, green, and red light, respectively, compared to the control (white light). By comparing the gene expression patterns with the growth and physiological responses of the grape plantlets, we were able to link the responses of the plants to light of different spectral wavelengths and the expression of particular sets of genes. Exposure to red and green light primarily triggered responses associated with the shade-avoidance syndrome (SAS), such as enhanced elongation of stems, reduced investment in leaf growth, and decreased chlorophyll levels accompanied by the expression of genes encoding histone H3, auxin repressed protein, xyloglucan endotransglycosylase/hydrolase, the ELIP protein, and microtubule proteins. Furthermore, specific light treatments were associated with the expression of a large number of genes, including those involved in the glucan metabolic pathway and the starch and sucrose metabolic pathways; these genes were up/down-regulated in ways that may explain the increase in the starch, sucrose, and total sugar contents in the plants. Moreover, the enhanced root growth and up-regulation of the expression of defense genes accompanied with SAS after exposure to red and green light may be related to the addition of 30 g/L sucrose to the culture medium of plantlets grown *in vitro*. In contrast, blue light induced the up-regulation of genes related to microtubules, serine carboxypeptidase, chlorophyll synthesis, and sugar degradation and the down-regulation of auxin-repressed protein as well as a large number of resistance-related genes that may promote leaf growth, improve chlorophyll synthesis and chloroplast development, increase the ratio of chlorophyll a (chla)/chlorophyll b (chlb), and decrease the ratio of carbohydrates to proteins in plants. Although exposure to red and green light seems to impose “shade stress” on the plantlets, growth under blue light is comparable to growth observed under white or broad-spectrum light.

## Introduction

Light quality plays an important role in plant growth by regulating a plethora of physiological activities. In the petunia, the elongation of the main stem is strongly inhibited when the plant is exposed to red light at irradiances of 70 and 150 μmol m^−2^ s^−1^ compared to that when it plant is exposed to white and blue light (Fukuda et al., 2016). However, exposure to a high proportion of blue light has also been shown to be effective in suppressing stem extension, growth of internodes and cell expansion, or division (Islam et al., 2012; Nanya et al., 2012; Terfa et al., 2013; Ouyang et al., 2015). Moreover, shoot growth in lettuce plants exposed to green light emitted by a light-emitting diode (LED; 510 nm) at 300 μmol m^−2^ s^−1^ was increased compared with plants exposed to white fluorescent light (Johkan et al., 2012). Leaf growth parameters, including specific leaf mass, thickness, and leaf density, were the lowest in *Alternanthera brasiliana* grown under red light (Macedo et al., 2011). In contrast, the number of leaves/plant and the thickness and area of the leaf blade in *A. brasiliana* (Macedo et al., 2011) and balloon flower (Liu et al., 2014a) were the greatest in plants grown under blue light than plants grown under other lights, but blue light did not affect total dry matter production in roses (Terfa et al., 2013). Exposure to green and red light also produces the smallest leaf area in *A. brasiliana* (Macedo et al., 2011); however, in bell pepper plants, the leaf area was greater under green covers (Casierra-Posada et al., 2014). After exposure to a short-duration of blue light from LEDs, the levels of shoot tissue pigments, glucosinolates, and mineral elements were obviously increased in sprouting broccoli (*Brassica oleacea* var. *italica*) (Kopsell and Sams, 2013). Light with a wavelength of 522 nm at 70 μmol m^−2^ s^−1^ reduced the fresh and dry masses of leaves and roots of *Lactuca sativa* and was associated with a reduced intensity of photosynthesis, reduced transpiration rate, and decreased stomatal conductivity compared with both red light (639 nm, 88 and 328 μmol m^−2^ s^−1^) and blue light (470 nm, 80 and 328 μmol m^−2^ s^−1^) (Golovatskaya and Karnachuk, 2015).

Light is the most important factor regulating plant growth and development *in vitro*, particularly the light in the spectral region that is involved in photosynthesis and in photomorphogenic responses (Dutta Gupta and Jatothu, 2013). In 1986, Chee first studied the effects of blue and red light on the morphogenesis of “Remaily Seedless” grapes cultured *in vitro* and showed that blue light was more effective at inducing shoot and root production (Chée, 1986). In the study by Heo et al. (2006), the fresh and dry weights of shoots of grape rootstock “Teleki5 BB” cultured *in vitro* were increased when plants were exposed to fluorescent lighting (control), red light, or a mixture of blue, and red light but were unaffected by blue-only radiation. Moreover, shoot length growth was significantly inhibited when the plants to a mixture of blue and red light, whereas the stems of plants grown under red light were more than twice as long as the stems of plants grown under fluorescent lighting. The positive effect of red light on shoot length growth has also been described by Poudel et al. (2007), who showed that “Hybrid Franc,” “Kadainou R-1,” and *Vitis ficifolia* var. “ganebu” grapes cultured *in vitro* produced the longest shoots with longer internodes under monochromic red light rather than those under other types of light. The highest chlorophyll content, leaf number per explant and number of stomata were observed on plants cultured under blue LEDs in all the genotypes studied. However, Poudel et al. (2007) also noted that different LEDs did not affect the rooting percentage of “Hybrid Franc,” but red LEDs yielded a higher rooting percentage and higher root numbers for the two other grape genotypes. Thus, the responses of grape plantlets to various LED light spectra are species-specific.

Previous microarray studies have identified differentially expressed genes (DEGs) in plants that are regulated by green light (Dhingra et al., 2006) and by the low red light/far-red light ratio (R:FR) (Reddy et al., 2013). Dhingra et al. (2006) confirmed that the expression of nuclear-encoded genes that encode components of the phytochrome system were affected by short, dim, single pulses of green light, and the levels of some plastid-encoded transcripts decreased after green light treatment, affecting seedling development during the critical process of early establishment. As shown in the studies of Reddy et al. (2013), after modifying the R:FR, DEGs in buds showed an enrichment of light signaling and hormone-related gene ontology terms and promoter motifs, most significantly the DEGs associated with the abscisic acid pathway. RNA-seq is superior to a microarray analysis in detecting low-abundance transcripts, and it has a broader dynamic range, allowing the identification of genetic variants and the detection of more DEGs with higher fold changes (Zhao et al., 2014). Furthermore, with the sequencing of more plant genomes and the rapid development of high-throughput sequencing, gene expression studies have been facilitated by the use of RNA-seq analysis. Ouyang et al. (2015) used RNA-seq to analyze the genetic mechanisms by which different light qualities regulate Norway spruce seedling growth and phytohormone levels and found that red light may promote stem growth by regulating the biosynthesis of gibberellic acids, whereas blue light may enhance plant defenses by increasing the levels of flavonoids, lignins, phenylpropanoids, and hormones, which may reduce the primary metabolites available for plant growth.

Although some researchers have reported the effects of various wavelengths of light on the growth and physiology of grape plantlets *in vitro*, the effects of light quality on grape transcription levels have not yet been scrutinized. In this study, “Manicure Finger” (*Vitis vinifera* L.) grape plantlets cultured *in vitro* were used to evaluate the effects of white, blue, green, and red light on plant growth and physiological characteristics. Moreover, using RNA-seq technology, the molecular basis of the response of the plants to various wavebands of light was explored at the transcriptional level. The results provide new insights into the mechanism by which grapes respond to light spectra and may contribute to the genetic improvement of grape growth *in vitro* under various types of light.

## Materials and methods

### Plant material

Leafy single-node cuttings (15 mm long) of the grape cultivar “Manicure Finger” (*Vitis vinifera* L.) were cultured *in vitro* on 3/4 Murashige and Skoog medium (40 ml per jar) (Murashige and Skoog, 1962) to which 0.35 mg/l indole butyric acid, 3% sucrose and 0.55% agar had been added. After a 24-h incubation in the dark, the cultures were maintained under blue, green, or red LED light fixtures for 40 days. A white fluorescent lamp (FL40D-EX/38, Huadian CO., China) emitting a wide range of wavelengths (from 350 to 750 nm) was used as the control light source. The spectral distributions of the LED lights (B, blue LEDs, peak at 440 nm; G, green LEDs, peak at 520 nm; and R, red LEDs, peak at 630 nm) were determined using a spectroradiometer (OPT-2000, ABDPE CO., Beijing, China). The half bandwidth of the peak wavelength was ± 10 nm. The photosynthetic photon flux density of each light was maintained at approximately 50 ± 5 μmol m^−2^ s^−1^, which was determined on the culture shelf at a vertical distance of 15 ± 1 cm from the light outside the glass jar (90–95% transmittance) using a quantum sensor (LI-250A, LI-COR, USA). The spacing between the glass jars was 5*5 cm. The light experiments were performed in an incubation room at a relative humidity of 80 ± 5% under a 12-h photoperiod at a temperature of 25 ± 2°C.

### Measurements of growth and physiological traits

After 40 days of illumination, the leaf number, plant height, and stem diameter were determined, and the leaf area and root characteristics were scanned on a flatbed scanner (Epson Expression 1680 1.0, Japan) and calculated using WinRHIZO (Regents Instruments, Quebec, Canada). The fresh and dry masses of each plant was determined, and the specific fresh leaf weight of all plantlets was measured. The chlorophyll (chl) contents of fresh leaves were measured by spectrophotometry using the method reported by Liu et al. (2011). The total sugar, soluble sugars, and starch contents were measured using the method described by Fairbairn (1954). The soluble protein contents were measured using the method reported by Vincent and Nadeau (1983). The chloroplast ultrastructure was observed under a scanning electron microscope (Barnes and Blackmore, 1984).

### Sample collection and RNA isolation

After 40 days, all leaf samples from 30 bottles of *V. vinifera* “Manicure Finger” from the four light treatment groups were collected and immediately frozen in liquid nitrogen to establish an mRNA library. Samples from each treatment were prepared as two replicates using the following procedure: Total RNA was isolated and extracted from the samples using Trizol reagent (Invitrogen Scientific, Inc., USA) according to the manufacturer's instructions. RNA integrity was confirmed by 1% agarose gel electrophoresis. A NanoDrop 1000 micro-ultraviolet-visible spectrophotometer (Thermo Fisher Scientific, Inc., USA) and an Agilent 2100 Bioanalyzer (Agilent Technologies, Inc., USA) were used to quantify the total RNA content and to determine its quality.

### RNA sequencing and reads mapping

The mRNA obtained from ~10 μg of total RNA was isolated, fragmented, converted to cDNA, and amplified by PCR according to the Illumina RNA-seq protocol (Illumina, Inc., USA). Sequence reads were generated using the Illumina Genome Analyzer II (Illumina) and Illumina HiSeq 2000 (Illumina) at the Beijing Genomics institution (ShenZhen, China) according to the manufacturer's recommendations. The FASTX toolkit (http://hannonlab.cshl.edu/fastx_toolkit/index.html) was used to clean the reads prior to mapping. The fastx_clipper program and the fastq_quality_trimmer were used to remove the Illumina adapter sequences and the low-quality bases from the ends of the reads. All the distinct clean reads were aligned to the grape genome database (ftp://ftp.jgi-psf.org/pub/compgen/phytozome/v9.0/Vvinifera/assembly/Vvinifera_145.fa.gz and ftp://ftp.jgi-psf.org/pub/compgen/phytozome/v9.0/Vvinifera/annotation/Vvinifera_145_transcript.fa.gz) using the SOAPaligner/SOAP2, with a maximum of two-base mismatches. We used the RPKM (Reads Per kb per Million reads) method (Mortazavi et al., 2008) to calculate the gene expression levels of each sample and used the RPKM values to compare differences in gene expression among the light treatments. If a gene encoded more than one transcript, the longest transcript was used to calculate the gene expression level and coverage.

### Screening of DEGs

This analysis screened the DEGs among the treatments and performed a Gene Ontology (GO) functional enrichment analysis and Kyoto Encyclopedia of Genes and Genomes (KEGG) pathway enrichment analysis of the DEGs. We applied the NOIseq method (Tarazona et al., 2011) to screen the DEGs between two groups. Genes were deemed significantly differentially expressed at a probability of ≥ 0.8 and an absolute value of log_2_ ratio ≥ 1 (the difference in expression was greater than 2). The Blast2GO program with default parameters was used to perform the GO functional enrichment analysis; after obtaining the GO annotations for the DEGs, the WEGO software was used to perform the GO functional classification of the DEGs. The results of the GO functional classification are displayed in three domains: Biological processes, cellular components, and molecular functions (Ye et al., 2006). In addition, the biological processes in which the DEGs were involved were defined by assigning the DEGs to metabolic pathways or signal transduction pathways using the KEGG annotation (Kanehisa et al., 2008). For the GO terms, we used corrected *P*-values < 0.05 to demonstrate a significant enrichment of the gene sets. KEGG pathways with threshold *Q*-values ≤ 0.25 were considered significantly enriched in the DEGs.

### Real-time quantitative PCR (qRT-PCR) validation of DEGs

We randomly selected 20 DEGs for the qRT-PCR analysis to validate the DEGs identified by RNA-seq. Actin1 (GRMZM2G126010) was used as the endogenous control in this study. Beacon designer software version 7.7 was used to design the corresponding primers, which are listed in Table 1. According to the standard protocol of the ABI7300 system, the amplification program was: 95°C for 30 s and 40 cycles of 95°C for 30 s, 60°C for 30 s, and 72°C for 30 s. A thermal denaturing step was used to generate melting curves to verify the specificity of the amplification. All reactions and negative controls were performed in triplicate biological replicates. In addition, the threshold cycles (Ct) of the triplicate reactions for each tested gene were averaged, and the values were normalized to the levels of the control actin1 gene product. We used the 2^−ΔΔCt^ method for the statistical analysis (Schefe et al., 2006).

**Table 1:** **The DEGs ID and primer sequences for qRT-PCR**.

**No**.	**Gene ID**	**Gene Names**	**Sense Primer**	**Anti-sense Primer**
1	GSVIVT01029183001	4-coumarate–CoA ligase	CCACAGAAAGAACCATAGATAAAG	AGCGGCATCAGAAACATTG
2	GSVIVT01036818001	Zeta-carotene desaturase	GGACACTGGCAACAACAAC	AAGGAGGAATGGAAGGAAGG
3	GSVIVT01001405001	Protein gigantea-like	TTTCTCTGTTGTTTCACTTCTTTG	ACCTGTCTCCATCCTTGTTG
4	GSVIVT01011072001	Glutamine synthetase	TCAGCAGTCAGAAGGTCATC	CCAGCACCACAGTAGTAAGG
5	GSVIVT01017279001	Polygalacturonase	GAAGTTGAGAGTAAGGGATATTGC	TATTGACACGAGAGGAAGAGC
6	GSVIVT01020069001	Alpha-amylase	AGGAGGGCTTTGGAATATGG	ACACTGCTGCTATCTTGACC
7	GSVIVT01020215001	Trehalose 6-phosphate synthase	AAGGAGCACAGGGAAAGATG	CATAATAGGCAGACTTCTCATAGC
8	GSVIVT01025544001	Circadian clock associated 1	TCCCTTGTGCCTGTATATTCTC	AGTGTTCTGCTTGACCTTCC
9	GSVIVT01033349001	Galacturan 1,4-alpha-galacturonidase	GGAGGAGATGTTAGTGATGTTAC	GCAGGTTGTGATGTAGATTGG
10	GSVIVT01035231001	Late elongated hypocotyl	ATCAGCAGTTGGTTCAGAGAC	TCGGTTGGTGGATTGAGTTC
11	GSVIVT01037343001	Hydroxymethylglutaryl-CoA lyase	GTTGCTGGTCTTGGAGGATG	GGCTATGGCTGTCTTGGAAC
12	GSVIVT01025088001	Pseudo-response regulator 7	GAGAGGAGCAGGAGATGTTG	TGATTAGCAGAAGAAGCAGTATTG
13	GSVIVT01028864001	Beta-carotene 3-hydroxylase	CACCAACTCCACCACTCAG	CCACCTCTTCCACTTCCTTAG
14	GSVIVT01012577001	Isoflavone 7-O-glucosyltransferase	AACACCTGCTCACTTACTACC	CACGGAGGCGATGTATGC
15	GSVIVT01020828001	Phytoene synthase	TAGGTGCGGTGAGGTCTG	TGTGAAGCATTAGGTCCATCC
16	GSVIVT01024400001	4-coumarate–CoA ligase	CGCTGCCGTTCTCCTCTG	GTTCTCGCCGTCCACCTG
17	GSVIVT01025800001	Cytochrome P450 98A2	CGCAACTGGCTGATAGGC	TGGCTGTCACTTCATCTTCTC
18	GSVIVT01026986001	Hydroquinone glucosyltransferase	TGTTTGTAGGGTTTGGGAGTG	GCATCGGCATCTTCAATAGC
19	GSVIVT01018606001	Elongation factor G	GCCAGCACCGAGTTCATC	GAAAGGGTCACAGCAAGGG
20	GSVIVT01032644001	Pseudo-response regulator 7	CACTGCTGTAAATGCCCAAG	TACTCCGCTCCTGCTTCC

### Data analysis

Each treatment (15 plantlets) was replicated three times to measure the growth traits (*n* = 45). Leaf samples from 15 plantlets in each treatment were used as one replication and three replications were applied to measure the physiological traits (*n* = 3). The data were subjected to analysis of variance (ANOVA), and differences between means were tested at the 5% level using Duncan's multiple range test. Computations were performed with SPSS for Windows, version 19.0 (SPSS Inc., Chicago, IL, USA). Thirty units per treatment were replicated two times in the RNA-seq experiment (*n* = 2). We used the RPKM method (Mortazavi et al., 2008) to calculate gene expression levels and used the RPKM values to compare the differences in gene expression among treatments. We applied the NOIseq method (Tarazona et al., 2011) to screen the DEGs between two treatments. Genes with a probability ≥ 0.8 and an absolute value log_2_ ratio ≥ 1 were deemed significantly differentially expressed. The DEGs from each treatment were uploaded to the website (http://bioinformatics.psb.ugent.be/webtools/Venn/) to plot Venn diagrams. A cluster analysis of the DEGs was conducted using TreeView for Windows, version 1.6.6 (http://taxonomy.zoology.gla.ac.uk/rod/rod.html).

## Results

### Effects of light quality on plantlet growth traits

After 40 days of incubation, significant differences were observed in the growth of leaves, shoots and roots among the plantlets grown under different spectral wavelengths of light. As shown in Table 2, the average values for the leaf area and fresh overground mass of plants exposed to white light were approximately 17.1 cm^2^ and 1.56 g/plant, respectively; these values were greater than the values obtained for plants exposed to monochromatic blue, green, or red light. The average leaf number, dry overground mass and specific fresh leaf weight of plants grown under white light were ~7, 122 mg/plant and 9.74 mg/cm^2^, respectively. These values were greater than the values obtained for plants grown under monochromatic red or green light, but were similar to the values for plants grown under blue light (Table 2). Furthermore, the average plant height, root length, root surface area, root volume, and root dry mass of the plantlets illuminated by green light were ~118 mm, 148 cm, 72 cm^2^, 2.83 cm^3^, and 57.58 mg/plant, respectively. These values were significantly greater than the values obtained for the plantlets grown under blue or white light and were similar to the values obtained for plants grown under red light (Table 2). Moreover, the plantlets illuminated by blue light attained the greatest average stem and root diameters (Table 2) of ~1.58 and 1.82 mm, respectively.

**Table 2:** **Effect of light quality on plantlets growth traits**.

**Light treatment**	**White**	**Blue**	**Green**	**Red**
Leaf area (cm^2^)	17.10±0.70a	14.50±1.09b	9.19±0.97c	7.38±0.90cd
Leaf number	7a	6ab	5c	5c
Plant height (mm)	86.25±10.08b	52.36±7.86c	118.16±10.67a	115.37±4.87a
Stem diameter (mm)	1.39±0.04b	1.58±0.12a	1.07±0.09c	0.95±0.13cd
Total length of root (cm)	85.60±5.33b	54.20±14.25c	148.73±29.40a	135.39±23.47a
Root area (cm^2^)	45.26±3.81c	30.58±6.69d	72.30±9.13a	65.22±9.85ab
Root diameter (mm)	1.68±0.07ab	1.82±0.14a	1.58±0.20b	1.54±0.14b
Root volume (cm^3^)	1.91±0.22b	1.38±0.25c	2.83±0.34a	2.52±0.48a
Fresh mass (g/plant)	1.56±0.13a	1.30±0.07b	1.33±0.11b	1.22±0.10bc
Aboveground dry mass(mg/plant)	122.73±14.03a	115.03±11.12ab	70.88±4.95c	72.53±11.11c
Root dry mass(mg/plant)	39.47±5.38bc	31.13±1.96d	57.58±7.49a	48.08±6.47ab
Specific leaf fresh weight(mg/cm^2^)	9.74±0.51a	9.00±0.49ab	6.97±0.81c	7.47±0.44c

*Mean ± Standard error (n = 45); Different letters indicate significant differences (Duncan's multiple range test, P ≤ 0.05)*.

### Effects of light quality on plantlet physiological traits

As shown in the results presented in Table 3, the total sugar content in grapes grown under green light was similar to the content in grapes grown under red light and increased to ~70 mg/g DW compared to the control, which was the highest content observed. However, the soluble sugar and starch contents in the leaves of plants grown under red light were significantly higher than those contents in plants grown under green light and showed approximately 2-fold and 4-fold higher than the control, respectively. The highest soluble protein content was observed in the leaves of plantlets exposed to blue light and ~24 mg/g FW higher than the content in plantlets grown under green light, which had the lowest leaf protein content. Furthermore, monochromatic light had more beneficial effects on the soluble sugar and starch contents in grape plantlets than compound light. Moreover, the chla, chlb, carotenoid, and total photosynthetic pigment contents in grape leaves from plants exposed to blue light were ~1.3-fold higher than the contents in the control, whereas the contents were significantly lower in plants that received green or red light treatment than the control, particularly in plants that received the red light treatment. Moreover, the highest chla/chlb levels were also observed in plants exposed to blue light; the white light treatment yielded the second highest levels, followed by those levels when exposed to the green and red light treatments.

**Table 3:** **Effect of light quality on plantlet primary and secondary metabolites**.

**Light treatment**	**White**	**Blue**	**Green**	**Red**
Total sugar content (mg/g DW)	409.94±14.53b	339.07±6.82c	471.69±16.81a	469.75±4.07a
Soluble sugar content (mg/g DW)	101.03±12.85d	124.82±4.09c	181.89±8.28b	210.96±8.01a
Starch content (mg/g FW)	0.99±0.10d	1.34±0.19bc	1.73±0.28b	4.31±0.32a
Soluble protein content (mg/g FW)	44.02±1.17b	53.03±4.63a	29.36±1.94c	41.98±3.91b
Chl a content (mg/g FW)	2.72±0.11b	3.56±0.12a	1.40±0.06c	0.93±0.05d
Chl b content (mg/g FW)	0.84±0.02b	1.06±0.04a	0.49±0.03c	0.33±0.02d
Carotenoid content (mg/g FW)	0.57±0.03b	0.76±0.03a	0.31±0.01c	0.20±0.01d
Total photosynthetic pigment content (mg/g FW)	4.13±0.16b	5.38±0.19a	2.20±0.10c	1.46±0.08d
Chl a/b	3.24	3.34	2.86	2.83

*Mean ± Standard error (n = 3); Different letters indicate significant differences (Duncan's multiple range test, P ≤ 0.05)*.

**Figure 1:**
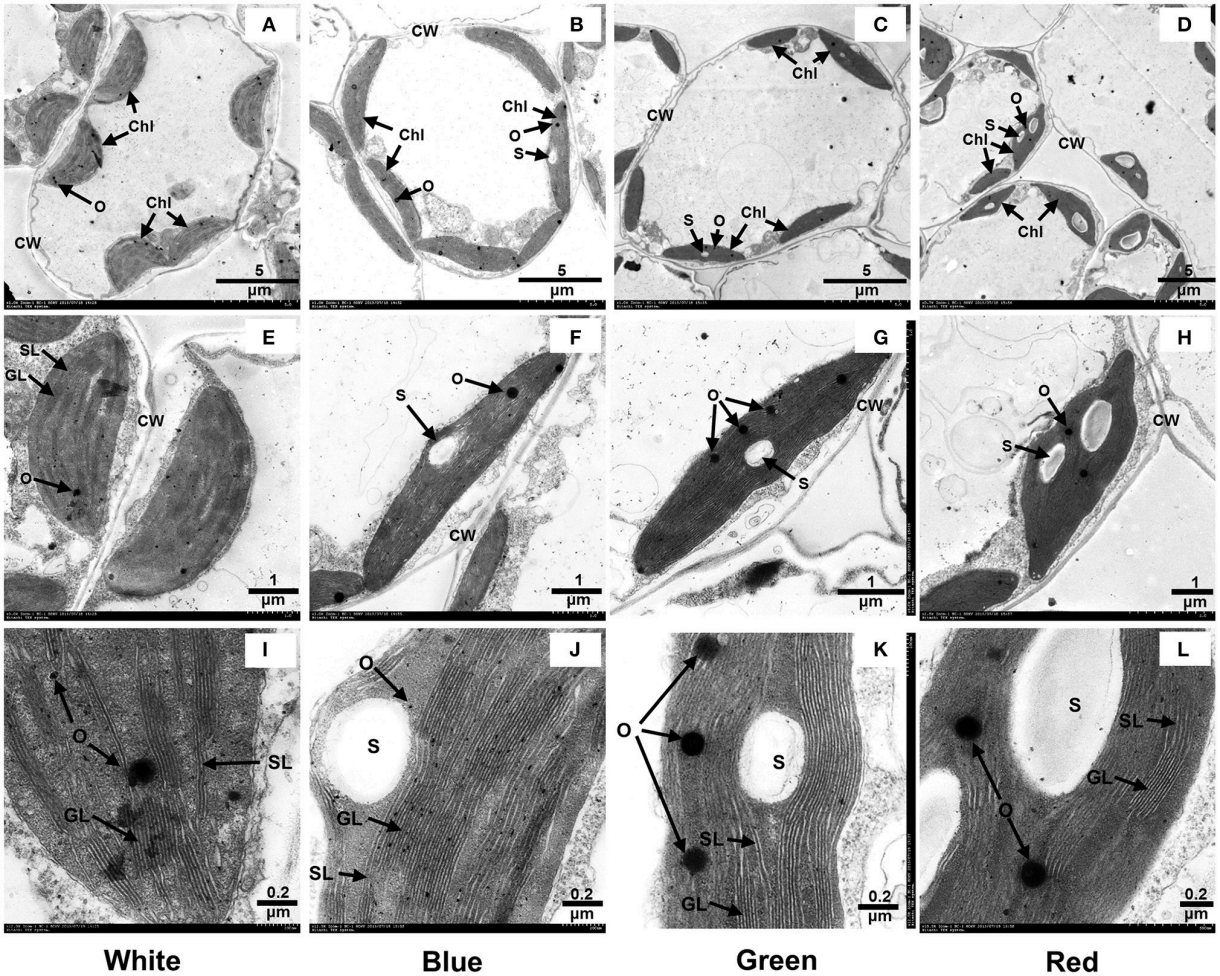
**Effect of light quality on the ultrastructure of leaf chloroplasts in grape plantlets grown *in vitro***. CW, cell wall; Chl, chloroplast; S, starch grain; O, osmophore; SL, stroma lamella; GL, grana lamellae. **(A)**, **(E)**, and **(I)** show the chloroplast structures in plants grown under white light; **(B)**, **(F)**, and **(J)** show the chloroplast structures in plants grown under blue light; **(C)**, **(G)**, and **(K**) show the chloroplast structures in plants grown under green light; and **(D)**, **(H)**, and **(L)** show the chloroplast structures in plants grown under red light.

We further observed the ultrastructure of chloroplasts to analyze the effects of light quality on chloroplast development. As shown in Figure 1, the chloroplasts in the mesophyll cells of plants that received white light treatment displayed a typical spindle shape and contained stromal lamellae, grana lamellae and a few osmophores (Figures 1A,E,I). In the mesophyll cells of the plants that received blue light, the chloroplasts had an elongated spindle shape and contained a few starch grains and osmophores, with tightly stacked lamellae of grana (Figures 1B,F,J). In the mesophyll cells of plants grown under green light, the chloroplasts were of non-uniform size and contained irregularly arranged lamellae and a few starch grains and osmophores (Figures 1C,G,K). The chloroplasts of plants grown under red light were misshapen due to the accumulation of large starch granules, which also limited the formation of stromal thylakoids (Figures 1D,H,L). Thus, white light promotes the normal development of chloroplasts in mesophyll cells, and blue light induces the formation of thylakoid lamellae in the chloroplasts. Although both red light and green light limit the normal development of chloroplasts, exposure to red light promotes the formation of starch grains.

### Effects of light quality on the transcriptome of grape plantlets

#### Statistics and sequencing quality assessment of the RNA-seq data

**Figure 2:**
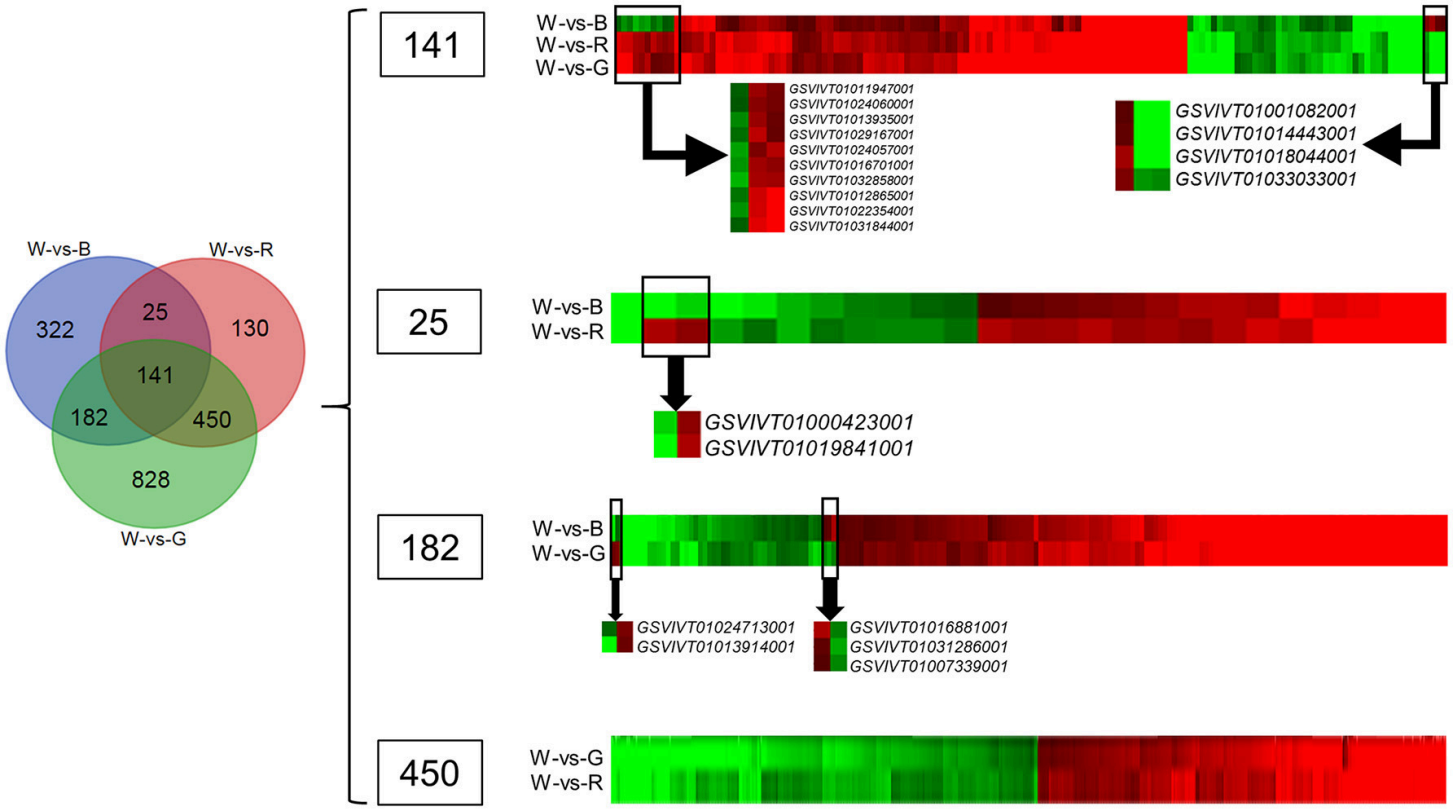
**Venn diagram and cluster analyses of DEGs in grape plants grown under red (R), blue (B), and green (G) lights compared with white light (W)**. Clustered heatmaps in the right show the overlapped DEGs in three or two comparison groups. The red color show the up-regulated genes in the plants grown under monochromatic lights compared with plants grown white light, while the green color show the down-regulated genes. The DEGs with different expression trend in each overlapped region were selected and shown alone below the clustered heatmap.

**Figure 3:**
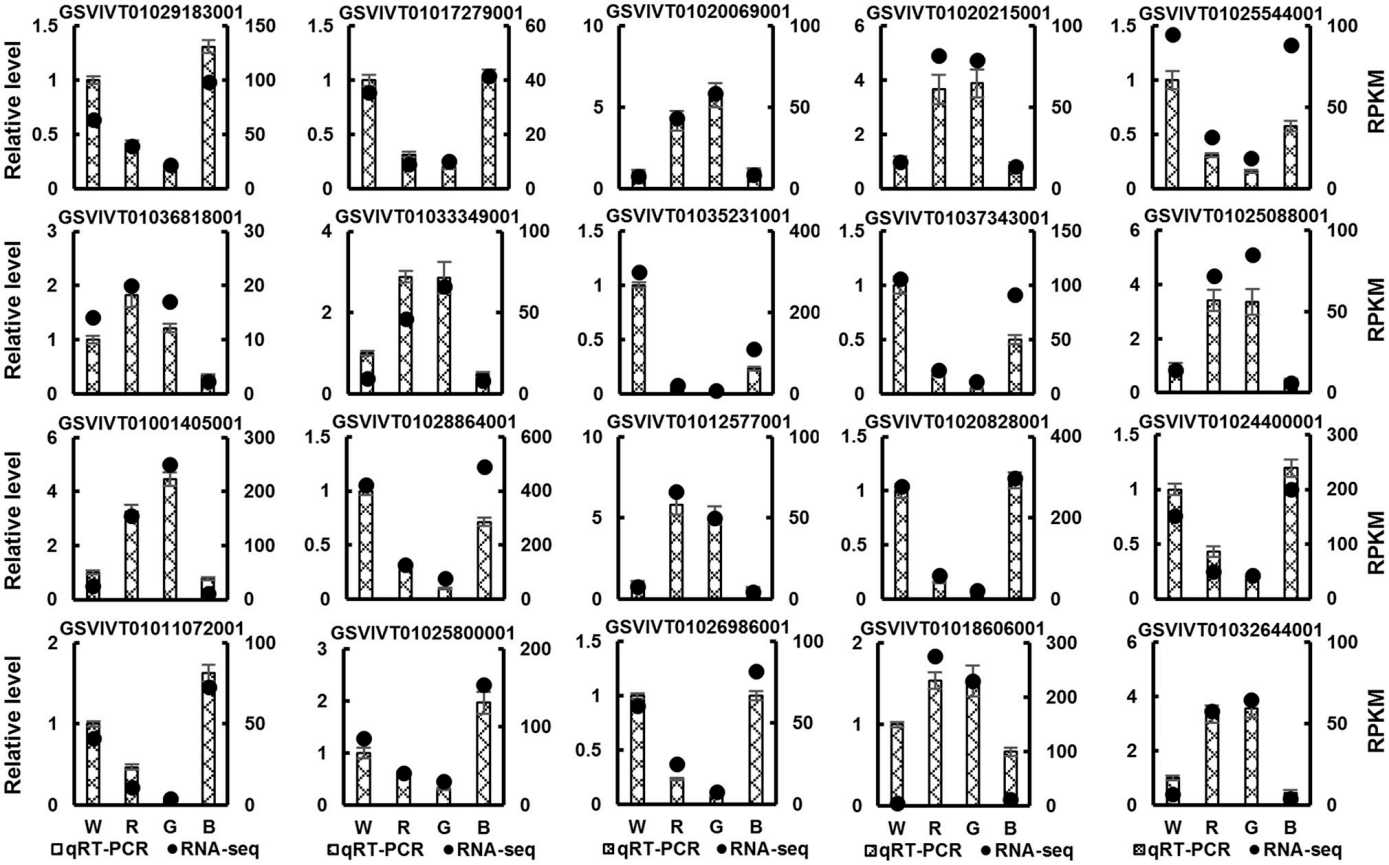
**Real-time quantitative PCR (qRT-PCR) validation of 20 randomly selected DEGs identified by RNA-seq in grape plants grown under white (W), red (R), green (G), and blue (B) light**. The histograms denote the qRT-PCR results. Mean value ± standard errors (SE). The black points denote the RNA-seq results. Mean RPKM value.

Based on the above-described growth and physiological features of plants exposed to varying wavelengths of light, we proposed that the expression of genes responsible for the observed changes in grape plantlets may have been differentially altered by the four light treatments. A transcriptome comparison was conducted to test our hypothesis. The transcriptome analysis data have been submitted to the National Center for Biotechnology Information (NCBI SAR: SRP076886). As shown in Supplement 1: Tables 1, 2 and Figures 1–5, from the 8 sequencing samples (each treatment contained two samples), we obtained an average of 12 million total sequencing reads of approximately 600 MB for each sample. Approximately 9 million reads from each sample were aligned to the grape reference genes and the reference genome, accounting for 75% of the total reads. Approximately 55% and 60% of the total reads perfectly matched the grape reference genes and the reference genome, respectively. At least 96% of the reads were high-quality reads (clean reads) and used for further analysis. The genes identified by sequencing saturation analysis represented ~80% of the sample and showed a uniform distribution in all loci, with high coverage. As shown in Supplement 4, the expression levels of 23,717 genes in each light-treated sample were calculated using the RPKM method. The obtained values of RPKM were used to analyze the differences in gene expression associated with each monochromatic light treatment compared to the gene expression of the control (white light treatment) (Supplements 5–7).

#### Results of the screen for the DEGs between treatments

As shown in Supplement 1: Figure 1, the correlations between the two biological replicates in the same treatment group were all greater than 90% [blue light (B): 98.57%, green light (G): 95.22%, red light (R): 90.22%, and white light (W): 98.57%], indicating the repeatability of the experiments. All clean reads were aligned to the grape genome; the comparative transcriptomic analysis was then performed to screen the DEGs using the NOIseq method (|log_2_ ratio| ≥ 1 and probability ≥ 0.8) (Supplement 1: Figure 6 and Supplements 5–7). As shown in Supplement 1: Figure 6, six hundred seventy genes were differentially expressed between B and W; of these DEGs, 418 genes were up-regulated and 252 genes were down-regulated in B compared with W (W-vs-B). Between G and W, 1601 genes were differentially expressed; of these DEGs, 925 genes were up-regulated and 676 genes were down-regulated in G compared with W (W-vs-G). Seven hundred forty-six genes were differentially expressed between R and W; of these DEGs, 455 genes were up-regulated, and 291 genes were down-regulated in R compared with W (W-vs-R).

#### GO analysis of DEGs in plants that received different light quality treatments

As shown in the results presented in Supplement 2 and Supplement 1: Figure 7, the GO analysis identified 944 (W-vs-B), 2462 (W-vs-G), and 989 (W-vs-R) DEGs that were enriched for the term “cellular component.” In W-vs-B, the DEGs were mainly enriched for the terms “cytoplasm,” “plastid,” “ribosome,” “microtubule cytoskeleton,” and “chromatin,” and genes associated with all these terms were up-regulated by B. In W-vs-G, the DEGs were primarily enriched for the terms “cytoplasm,” “plastid,” “thylakoid,” and “chromatin”; genes associated with many of these terms were up-regulated by G, whereas plastid-related genes were ambiguous between G and W. In W-vs-R, the DEGs were primarily enriched in “cytoplasm,” “plastid,” “chromatin,” and “cell wall”; genes in the terms “cell wall” and “chromatin” were up-regulated by R, whereas genes in the “cytoplasm” term were ambiguous between R and W.

As shown in Table 4, Supplement 2 and Supplement 1: Figure 7, the analysis identified 527 (W-vs-B), 1361 (W-vs-G), and 594 (W-vs-R) DEGs that were enriched for the term “molecular function.” In W-vs-B, the DEGs were mainly enriched for the terms “motor activity” and “carboxypeptidase activity,” both of which were up-regulated by B. In W-vs-G, the DEGs were mainly enriched in “protein binding” and “structural molecule activity”; the number of genes enriched in “protein binding” in G was slightly higher than the number enriched in W, whereas most of the genes associated with “structural molecule activity” were up-regulated by G. In W-vs-R, the DEGs were mainly enriched in the categories “hydrolase activity, acting on glycosyl bonds” and “transferase activity, transferring hexosyl groups,” both of which were up-regulated by R.

**Table 4:** **Molecular_function and Biological_process enrichment analyses for DEGs between different light qualities**.

**GO domain**	**GO terms ID**	**Gene Ontology term**	**Corrected *P*-value (<0.05)**	**UP-regulated DEGs**	**Down-regulated DEGs**	**Total DEGs**
Molecular_function		**W-vs-B**				
	GO: 0003774	Motor activity	0.00022	12	0	12
	GO: 0004180	Carboxypeptidase activity	0.00178	9	0	9
		**W-vs-G**				
	GO: 0005515	Protein binding	0.00218	50	37	87
	GO: 0005198	Structural molecule activity	0.01232	35	6	41
		**W-vs-R**				
	GO: 0016798	Hydrolase activity, acting on glycosyl bonds	0.00323	24	8	32
	GO: 0016758	Transferase activity, transferring hexosyl groups	0.02418	17	5	22
Biological_process		**W-vs-B**				
	GO: 0034728	Nucleosome organization	0.00187	9	0	9
	GO: 0071824	Protein-DNA complex subunit organization	0.00231	9	0	9
	GO: 0043933	Macromolecular complex subunit organization	0.01025	15	0	15
	GO: 0007017	Microtubule-based process	0.02412	11	0	11
	GO: 0006779	Porphyrin-containing compound biosynthetic process	0.04296	8	0	8
		**W-vs-G**				
	GO: 0034728	Nucleosome organization	0.00501	11	2	13
	GO: 0006457	Protein folding	0.00579	7	0	7
	GO: 0048522	Positive regulation of cellular process	0.00588	3	5	8
	GO: 0071824	Protein-DNA complex subunit organization	0.00674	11	2	13
	GO: 0005982	Starch metabolic process	0.02788	6	3	9
		**W-vs-R**				
	GO: 0044042	Glucan metabolic process	0.01167	12	4	16
	GO: 0034728	Nucleosome organization	0.02295	6	2	8
	GO: 0071824	Protein-DNA complex subunit organization	0.02751	6	2	8

As shown in Table 4, Supplement 2 and Supplement 1: Figure 7, the “biological process” category included 815 (W-vs-B), 2344 (W-vs-G) and 962 (W-vs-R) DEGs. In W-vs-B, the DEGs were enriched in “nucleosome organization,” “protein-DNA complex subunit organization,” “macromolecular complex subunit organization,” “microtubule-based process,” and “porphyrin-containing compound biosynthetic process,” and all these DEGs were up-regulated in B. In W-vs-G, the DEGs were enriched in “nucleosome organization,” “protein folding,” “protein-DNA complex subunit organization,” “starch metabolic process,” and “positive regulation of cellular process”; with the exception of “positive regulation of cellular process,” which was primarily up-regulated by G, these terms were all mainly up-regulated by W. In W-vs-R, the DEGs were enriched in “glucan metabolic process,” “nucleosome organization,” and “protein-DNA complex subunit organization,” all of which were up-regulated by R.

#### KEGG pathway analysis of DEGs in the different treatments

In plants, various genes are coordinated to perform their biological functions, and pathway analyses help researchers understand the biological mechanisms. In the present study, a KEGG pathway enrichment analysis was performed for W-vs-B, W-vs-G, and W-vs-R (Table 5 and Supplement 3). In W-vs-B, 407 DEGs were annotated and were enriched in “other glycan degradation” and “porphyrin and chlorophyll metabolism,” which were mainly up-regulated by B. In W-vs-G, 994 DEGs were annotated and were enriched in “ribosome,” “circadian rhythm,” “carbon fixation,” “phenylpropanoid biosynthesis,” “Starch and sucrose metabolism,” and “Glycine, serine and threonine metabolism.” Eighty-five percent of the genes in “ribosome” were up-regulated by G, the number of up-regulated genes in “circadian rhythm” in W and G were equal, the number of up-regulated genes in “carbon fixation” was slightly higher in W than in G, and the number of up-regulated genes in the remaining pathways was all slightly higher in G than in W. In W-vs-R, 451 DEGs were annotated and were enriched in “circadian rhythm”; the number of up-regulated genes in this category was similar in W and R.

**Table 5:** **Significantly enriched pathways of DEGs between different light qualities**.

**Pathway ID**	**Pathway**	**DEGs with pathway annotation**	***Q*-value (<0.05)**	**UP-regulated DEGs**	**Down-regulated DEGs**
	**W-vs-B**				
	Total DEGs with pathway annotation	407 (100%)			
ko00511	Other glycan degradation	12 (2.95%)	0.04457	9	3
ko00860	Porphyrin and chlorophyll metabolism	8 (1.97%)	0.04457	8	0
	**W-vs-G**				
	Total DEGs with pathway annotation	994 (100%)			
ko03010	Ribosome	57 (5.73%)	8.09E-09	48	9
ko04712	Circadian rhythm - plant	28 (2.82%)	2.52E-07	14	14
ko00710	Carbon fixation in photosynthetic organisms	16 (1.61%)	4.01E-03	6	10
ko00940	Phenylpropanoid biosynthesis	45 (4.53%)	8.31E-03	25	20
ko00500	Starch and sucrose metabolism	43 (4.33%)	1.32E-02	29	14
ko00260	Glycine, serine and threonine metabolism	15 (1.51%)	3.36E-02	9	6
	**W-vs-R**				
	Total DEGs with pathway annotation	451 (100%)			
ko04712	Circadian rhythm - plant	17 (3.77%)	1.34E-05	8	9

#### Venn diagram analysis and cluster analysis of DEGs among the treatments

The Venn diagram analysis (Figure 2) of the DEGs in W-vs-B, W-vs-R, and W-vs-G identified 141 overlapping genes in the three comparison groups; these genes may be associated with basic processes that are regulated by light quality. According to the clustered heatmap (Figure 2), most of these genes displayed similar expression trends, only 14 of these DEGs showed differential expression patterns; four, including the genes for heme-binding protein (*GSVIVT01001082001*), dynein light chain LC6 (*GSVIVT01014443001*), chloroplast ELIP early light-induced protein (*GSVIVT01018044001*), and phosphoric diester hydrolase (*GSVIVT01033033001*), were up-regulated by B but inhibited by R and G, whereas 10 genes were down-regulated by B and up-regulated by R and G, RAV-like factor (*GSVIVT01011947001*), phosphoric ester hydrolase (*GSVIVT01024060001*), EREBP-like factor (*GSVIVT01013935001*), xyloglucan endotransglucosylase (*GSVIVT01029167001*), molecular chaperone DnaJ (*GSVIVT01024057001*), auxin-repressed 12.5-kDa protein (*GSVIVT01016701001*), defense response proteins (*GSVIVT01032858001* and *GSVIVT01031844001*), and unnamed protein products (GSVIVT01012865001 and *GSVIVT01022354001*), compared with W (Figure 2 and Table 6).

**Table 6:** **The KEGG or GO or Blast nr analysis for DEGs from the clustered heatmap analyses**.

**Gene ID**	**log_2_ Ratio (B/W)**	**log_2_ Ratio (R/W)**	**log_2_ Ratio (G/W)**	**Definition (KEGG or GO or Blast nr)**
**W-vs-B and W-vs-R and W-vs-G**				
*GSVIVT01001082001*	1.053	−3.555	−5.343	Heme-binding protein 2-like
*GSVIVT01014443001*	1.167	−3.332	−5.655	Dynein light chain LC6
*GSVIVT01018044001*	1.917	−4.432	−4.503	Chloroplast ELIP early light-induced protein
*GSVIVT01033033001*	1.493	−1.818	−1.658	Phosphoric diester hydrolase activity
*GSVIVT01011947001*	−1.131	1.849	1.406	RAV-like factor
*GSVIVT01024060001*	−1.061	1.623	1.379	Phosphoric ester hydrolase activity
*GSVIVT01013935001*	−1.638	1.833	1.261	EREBP-like factor
*GSVIVT01029167001*	−1.307	2.235	1.234	Xyloglucan endotransglucosylase protein
*GSVIVT01024057001*	−1.942	1.484	2.139	Molecular chaperone DnaJ
*GSVIVT01016701001*	−1.781	1.915	1.699	Auxin-repressed 12.5 kDa protein-like
*GSVIVT01032858001*	−2.183	1.991	1.905	Defense response
*GSVIVT01012865001*	−1.399	2.450	2.948	Unnamed protein product
*GSVIVT01022354001*	−1.753	2.474	3.028	Unnamed protein product
*GSVIVT01031844001*	−1.129	2.686	3.263	Defense response
**W-vs-B and W-vs-R**				
*GSVIVT01000423001*	−2.520	1.657	−	Calmodulin
*GSVIVT01019841001*	−3.030	2.044	−	Thaumatin-like protein
**W-vs-B and W-vs-G**				
*GSVIVT01024713001*	−1.227	−	1.467	Asparagine synthetase
*GSVIVT01013914001*	−3.676	−	1.297	EREBP-like factor
*GSVIVT01016881001*	2.045	−	−1.519	Pyridoxal phosphate phosphatase
*GSVIVT01031286001*	1.144	−	−2.031	ABC transporter
*GSVIVT01007339001*	1.016	−	−1.606	Transcription factor MYC2

The Venn diagram analysis also identified 25 DEGs that overlapped in W-vs-B and W-vs-R. Twenty-three of these DEGs showed similar expression patterns in B and R, and only two, the genes encoding calmodulin (*GSVIVT01000423001*) and thaumatin-like protein (*GSVIVT01019841001*), were down-regulated by B and up-regulated by R compared with W (Figure 2 and Table 6).

One hundred eighty-two genes overlapped between W-vs-B and W-vs-G were identified by Venn diagram analysis; of these DEGs, 177 showed similar expression patterns in W-vs-B and W-vs-G, whereas two, asparagine synthetase (*GSVIVT01024713001*) and EREBP-like factor (*GSVIVT01013914001*), were induced by B but inhibited by G compared with W. Three of these genes, pyridoxal phosphate phosphatase (*GSVIVT01016881001*), ABC transporter (*GSVIVT01031286001*) and transcription factor MYC2 (*GSVIVT01007339001*), were up-regulated by G but down-regulated by B compared with W (Figure 2 and Table 6).

Four hundred fifty genes overlapped in W-vs-G and W-vs-R were identified by Venn diagram analysis, all of which showed similar expression patterns in G and R compared with W (Figure 2 and Table 6).

#### Confirmation of the RNA-seq results using qRT-PCR

To validate the RNA-seq results, 20 DEGs were randomly selected, and a qRT-PCR analysis of these genes was performed (Figure 3). Although the relative expression of these genes was different in the RNA-seq and qRT-PCR data, the expression patterns of these genes were similar in the data obtained using the two methods, confirming the accuracy of the RNA-seq results.

## Discussion

### Transcriptional responses of grape plantlets to various qualities of light *in vitro*

In our study, the growth of the leaves and the total dry weight of plants grown under monochromatic green and red light were inferior to plants grown under monochromatic blue light or white light (compound light) (Table 2). According to previous studies, the expression of histone H1, H2A, H2B, H3, and H4 genes is positively correlated with cell division during leaf and root growth (Terada et al., 1993; Prymakowska-Bosak et al., 1996; Bilgin et al., 1999; Rodriguez et al., 2014). However, as shown in Table 4 and Supplement 2 in our study, all histone DEGs (H2A, H2B, and H4) involved in the process of protein-DNA complex organization were up-regulated by blue light. Two H3 genes were down-regulated by green light, and two histone DEGs (H3 and H4) were down-regulated by red light, although the other histone DEGs (H1, H2A, H2B, and most of H4) were up-regulated by these two light treatments. Thus, the expression of the histone H3 gene may have been the primary factor that positively regulated the leaf growth and total dry weight of grape plantlets. However, the leaf area of plants grown under monochromatic blue light was greater than the leaf area of plants grown under monochromatic red or green light (Table 2), which may be related to the expression of tubulin-, dynein-, kinesin-, and microtubule-binding-protein-related genes, all of which were significantly up-regulated by blue light (Table 4 and Supplement 2). Microtubules are involved in cell division, the maintenance of cell shape and function, and cell movement; together with cellulose microfibrils, microtubules also play an important role in the growth and development of the secondary cell walls of plants (Martin et al., 2002; Cosgrove, 2005; Pesquet et al., 2010). Plant tubulin is also an important component of the plant cytoskeleton, which is needed to maintain cell morphology. In addition, microtubules are needed for cell organelle movement and the intracellular translocation of substances (Tiwari et al., 1984; Derksen et al., 1990; Volkmann and Frantis, 1999). Therefore, we propose that the blue light-induced increase in the expression of microtubule-associated proteins promoted cell division and leaf growth.

The blue light-induced expression of microtubule-associated proteins also resulted in increased diameters of the stems of grape plantlets compared to other treatments (Table 2). As shown in previous studies, the distribution of microtubules corresponds to the sites of deposition of vessel wall microfibrils, allowing microtubules to promote cell wall thickening (Pickett-Heaps, 1967; Wightman and Turner, 2008). Similarly, plant tubulin was also shown to be related to the synthesis of cellulose and lignin (Gardiner et al., 2003; Spokevicius et al., 2007; Dornelas and Mazzafera, 2010), which promotes the formation of the secondary cell wall. Thus, the expression of microtubule-associated proteins in plants exposed to the blue light treatment induces the formation of secondary cell walls in the stem, causing the plant stem to thicken.

Plant height and root growth were reduced in response to the blue light treatment compared with the red or green light treatment (Table 2), possibly due to the blue light-induced increase in the expression of serine carboxypeptidase-like protein (SCLP) genes (Table 4) and decrease in the expression of the auxin-repressed protein gene (Table 6). As shown in the study by Bienert et al. (2012), overexpression of the SCP genes *NtSCP1 and NtSCP2* in transgenic tobacco plants limits cell expansion and extension, decreases the size of flowers and fruits, and limits hypocotyl elongation in etiolated seedlings. Thus, blue light likely inhibits plant stem elongation by up-regulating the expression of the SCPL gene. In the study by Park and Han (2003), the expression of the ARP gene was down-regulated or even completely abolished in the presence of high concentrations of exogenous auxin. Thus, in plants grown under blue light, significant concentrations of auxin accumulate and inhibit the expression of the *ARP* gene; however, because the concentration of auxin exceeds the optimal concentration for root and stem growth, root and stem elongation is inhibited.

In contrast to blue light, red light and green light notably promoted the expression of the auxin inhibitor protein gene (Table 6), suggesting that the auxin concentrations are low in red light- and green light-treated plants, a condition that is suitable for plant stem and root length growth. Moreover, the expression of the xyloglucan endotransglycosylase/hydrolase gene (XTH) (Table 6) was similar to the expression of auxin-repressed protein in plants grown under all light treatments. Cell wall loosening is the foundation of rapid cell expansion, and XTH is a key factor affecting the relaxation and extension of the cell wall (Cosgrove, 2000). As shown in the study by Zhang et al. (2012), the growth rate of *OsXTH11* transgenic rice plants is higher than the growth rate of wild type plants. Xyloglucan is mainly distributed in the primary cell wall area (McCann et al., 1990; Hayashi, 2003). Therefore, compared to the secondary cell wall formation induced by blue light, exposure to red light and green light appears to play a major role in the elongation growth of the primary cell wall, and thus, plants exposed to red or green light had longer, more slender stems.

### Transcriptional responses of genes associated with chlorophyll, sucrose, starch, and protein metabolism in plants exposed to various types of light

In the study by Wang et al. (2009), the chlorophyll contents in the leaves of plants exposed to white, violet or blue light were significantly higher than the contents in the leaves of plants exposed to yellow, green, or red light. Voskresenskaya (1972) reported that the chlorophyll content in barley leaves decreased and an abnormal chloroplast structure was observed following red light irradiation, whereas under blue light irradiation, the chlorophyll content was relatively stable, and the abnormalities and deactivation of chloroplasts caused by red light were reversed. In our study, the photosynthetic pigment (chlorophyll) contents in the leaves of grape plantlets grown under blue light were significantly higher than the contents in plantlets grown under white, green or red light (Table 3). Furthermore, blue light notably promoted the development of chloroplast and thylakoid lamellae compared to red and green light (Figure 1). These effects may be related to the blue light-induced up-regulation of the expression of genes related to chlorophyll synthesis. The protein products of some of these genes are located in the plastids, ribosomes, and thylakoids, where they participate in the biosynthesis of porphyrin-containing compounds. These gene products include glutamate-1-semialdehyde 2, 1-aminomutase (HemL), hydroxymethylbilane synthase (HemC), uroporphyrinogen decarboxylase (HemE), coproporphyrinogen III oxidase (HemF), protoporphyrinogen oxidase (HemY), magnesium-protoporphyrin-O-methyltransferase, protochlorophyllide reductase, and chlorophyll(ide) b reductase (Tables 4, 5 and Supplement 1: Figure 8). In addition, the increased ratio of chla/chlb observed in plants grown under blue light may be related to the chlorophyllide/chlorophyll reductase that catalyzes the conversion of chlorophyllide/chlorophyll b into chlorophyllide/chlorophyll (Table 5 and Supplement 1: Figure 8). Moreover, the blue light-induced increase in the expression of the genes encoding the heme-binding 2-like protein and chloroplast ELIP early light-induced protein (Table 6) may increase chlorophyll accumulation and promote chloroplast development. In previous studies, ELIP expression in pea and barley seedlings was more strongly induced by blue light than that by red and far red light (Adamska et al., 1992; Adamska, 1995). The lack of ELIP reduced the chlorophyll and zeaxanthin contents and decreased pigment synthesis in mature leaves, indicating that ELIP may play a key role in the synthesis and stability of those natural pigments (Rossini et al., 2006). Finally, the intracellular transport of pigment granules in leaves is related to the plant microtubule-associated proteins dynein and kinesin (Gyoeva, 2005); these proteins have a significant effect on the accumulation of photosynthetic pigments in leaves and on the development of chloroplasts. In our study, the expression of these microtubule proteins (Table 4 and Supplement 2) was significantly increased by blue light compared with the control, while the expression of dynein light chain (LC6) microtubule protein was significantly decreased by red or green light (Table 6). Therefore, the photosynthetic pigment contents and chloroplast development in the leaves of grape plantlets were notably promoted by blue light but were inhibited by green or red light (Table 3 and Figure 1).

High sucrose concentrations in the culture medium of plants grown *in vitro* can cause starch and sucrose accumulation in the leaves, inhibiting the activity of the Rubisco enzyme (Hdider and Desjardins, 1994) and reducing chlorophyll synthesis (Neumann and Bender, 1987; Kirdmanee et al., 1992). Consequently, photosynthetic carbon assimilation is decreased. In our study, the highest sucrose accumulation was observed in red- and green light-treated plants, and significant amounts of starch accumulated in red light-treated plants. Thus, chlorophyll synthesis and chloroplast development were noticeably inhibited by the red and green light (Tables 4–6 and Figure 1). In response to the blue and white light treatments, starch and sucrose accumulation were limited, and chlorophyll synthesis and chloroplast development were promoted.

One possible reason for the increased sucrose accumulation observed in grape plantlets exposed to red and green light is the better root growth of these plants (Table 2). A more robust root system would promote the absorption and accumulation of excess sucrose from the culture medium, thereby limiting chlorophyll synthesis and chloroplast development. For the plants grown under green light, the carbon fixation pathway were significantly inhibited (Table 5), the down-regulated genes participated in C3 carbon fixation pathway encoding fructose-bisphosphate aldolase, fructose-1,6-biphosphatase, phosphoenolpyruvate carboxylase, glutamate-glyoxylate aminotransferase, sedoheptulose-biphosphatase, and glyceraldehyde-3-phosphate dehydrogenase, while the up-regulated genes only encoding the triosephosphate isomerase and ribulose bisphosphate carboxylase. Thus, the main assimilation source of green light-treated plants was absorption from the culture medium by the roots rather than fixation by photosynthesis.

Another possible reason for the increased sucrose accumulation observed in plants grown under red and green light compared to plants grown under blue or white light is that blue light promotes the translocation and utilization of starch and sucrose from chloroplasts, whereas red light seems to inhibit the translocation process (Sæbø et al., 1995). Blue light has been shown to produce a low carbohydrate to protein ratio in plants, whereas red light seems to produce an opposite trend (Voskresenskaya, 1972). These conclusions are consistent with the experimental results from our study (Table 3 and Figure 1). The coordinated up- or down-regulation of large numbers of related genes could explain this result. The expression of the genes encoding hexosaminidase, α-L-fucosidase, and β-galactosidase, all of which are associated with the polysaccharide degradation pathway, was significantly up-regulated by blue light (Table 5), thereby promoting the hydrolysis and metabolism of photosynthetic products (starch and sucrose). In addition, the blue light-induced expression of a large number of microtubule-associated proteins (Table 4 and Supplement 2) also had significant effects on the carbohydrate to protein ratio. On one hand, protein is the main component of microtubules; on the other hand, microtubules, in combination with kinesin and dynein, promote the translocation of intracellular substances (Gyoeva, 2005). Furthermore, the up-regulation of serine carboxypeptidase-like protein in plants that received the blue light treatment (Table 4) is expected to have a positive effect on the synthesis of plant secondary metabolites (Lehfeldt et al., 2000) and to subsequently assist in the translocation and utilization of starch and sucrose.

Interestingly, according to the results of the physiological assessment (Table 3), significant differences were observed in the starch and soluble sugar contents in the leaves of plants exposed to green and red light, although the total sugar content in these plantlets had no difference. This finding was due to the effects of these two kinds of light on the expression of genes related to sugar and starch metabolism (Tables 4, 5 and Supplements 2, 3). Although red light up-regulated the expression of genes related to glycosyl hydrolase and hexosyl transferase activity, it significantly inhibited the expression of β-amylase, peptide/histidine transporter, endoglucanase, granule-bound starch synthase, and digalactosyl 2-acylglycerol synthase genes (Tables 4, 5 and Supplements 2, 3). Although the expression of some of the β-amylase genes was significantly inhibited in plants treated with green light, the expression of one β-amylase gene (*GSVIVT01014681001*) and other starch and sucrose metabolism genes, such as the maltose excess protein/maltose transport protein, 4-α-glucose transferase and α-amylase genes, was significantly increased (Tables 4, 5 and Supplements 2, 3). In the study by Scheidig et al. (2002), reduced β-amylase expression may lead to the excess accumulation of starch in leaves. Maltose is the main product of β-amylase-mediated digestion of starch and is the main carbon source utilized by plants at night (Weise et al., 2004; Lloyd et al., 2005). As shown in the study by Chapman et al. (2013), MEX1 primarily transports maltose to the cell matrix from the plastid/chloroplast at night and promotes the hydrolysis of starch by β-amylase. Thus, we speculate that green light acts in this metabolic process as a type of signal of nighttime that induces *MEX1* gene expression, thereby promoting starch digestion and maltose transport and significantly reducing the starch and soluble sugar contents in the leaves compared to plants grown under red light.

### Shade-avoidance syndrome of grape plantlets grown under red and green light treatment

Plants have evolved various phenotypically plastic traits to help sustain light capture and avoid becoming overgrown and thus shaded by neighboring plants. These traits comprise the so-called shade-avoidance syndrome (SAS) and include enhanced elongation of stems and petioles, upward leaf movement (hyponasty), reduced investment in other organs, such as roots and leaf blades, decreased leaf chlorophyll levels, decreased maximal photosynthesis, and reduced expression of defense genes to release resources for shade-avoidance reactions (Casal, 2012; Pierik and de Wit, 2013). In our study, the growth and physiological characteristics of plantlets exposed to red and green light treatments included characteristics that are normally associated with shade avoidance, with the exception of root growth and the expression of defense genes. In response to red and green light, the expression of a large number of genes involved in plant defense was up-regulated, including the genes encoding histones H1, H2A, H2B, H3, and H4 (Ascenzi and Gantt, 1997; Bilgin et al., 1999; Klosterman et al., 2003; Isaac et al., 2009), auxin-repressed protein (Aharoni et al., 2002), xyloglucan endotransglucosylase/hydrolase (Choi et al., 2011), EREBP-like factor (Dietz et al., 2010), RAV-like factor (Zhao et al., 2008), the molecular chaperone DnaJ (Wang et al., 2004), and other defense-response-related proteins (Tables 4–6). In addition, the expression of genes encoding ribosomal proteins, which are up-regulated during defense resistance in plants (Sahi et al., 2006; Liu et al., 2014b; Vélezbermúdez and Schmidt, 2014), was significantly increased by green light (Table 5 and Supplement 3). In our study, the addition of exogenous sucrose (30 g/L) to the culture medium provided sufficient resources for plant defense resistance; therefore, the plants had no need to decrease the expression of defense-related genes to release resources for shade-avoidance reactions. Moreover, the robust growth of roots in plants exposed to red or green light promoted the absorption of sucrose from the medium. However, the growth and physiological characteristics of plantlets that received blue light treatment were similar to the characteristics of plants grown under white light; these plants did not display SAS but instead displayed “sun plant” characteristics. Thus, grape plantlets grown under the light spectrum without blue light *in vitro* would express SAS and would not grow well.

## Conclusions

In summary, this study provides integrated insights into the responses of grape plantlets to various light wavelengths, including white, blue, green, and red light. First, compared with white light, red, and green light treatments were associated with the expression of a large number of genes, such as those encoding histone H3, auxin-repressed protein, xyloglucan endotransglycosylase/hydrolase, ELIP protein, microtubule-associated proteins, and the genes involved in glucan, starch, and sucrose metabolic pathway. The up- or down-regulation of these genes could explain the enhanced elongation of stems and roots, the reduced investment in leaves, the decreased leaf chlorophyll levels, and the increased starch, sucrose and total sugar contents observed in the treated plantlets. Second, a large number of defense genes, including EREBP-like factor, RAV-like factor, molecular chaperone DnaJ, and other defense genes, were up-regulated by the red and green light treatments and accompanied by SAS. Finally, blue light up-regulated the expression of genes related to microtubules, serine carboxypeptidase activity, chlorophyll synthesis, and sugar degradation and down-regulated the expression of auxin-repressed protein and a large number of resistance-related genes; together, these changes in gene expression promoted leaf growth, improved chlorophyll synthesis and chloroplast development, increased the chla/chlb ratio, and decreased the carbohydrate to protein ratio, indicating that blue light is an important part of compound white light and that grape plantlets would not grow well without blue light. These findings contribute to a better understanding of the molecular basis of the responses of grape plants to various light wavebands, yield new insights into the mechanisms by which grapes respond to light quality, and are helpful in designing methods for the genetic improvement of grape growth *in vitro* in response to various light spectra.

However, further research should be conducted to extend the findings described above, for example, to measure the photosynthesis of grape plantlets and to study the effects of various spectral wavelengths on plants in the presence or absence of sucrose in the medium. Future experiments may seek the answers to a number of important questions: Are the effects of light quality on plants permanent? What portion of the blue spectrum is actually needed for the growth of grape plantlets *in vitro*? Do the physiological and transcriptional responses of grape plantlets grown *in vivo* and *in vitro* differ?

## Author contributions

CL performed the experiments, analyzed the data, and wrote and revised the manuscript. RD, LW, and MK helped conduct the experiments. SC helped analyze the data. JT and ZX designed the study and critically edited the manuscript. All authors approved the final manuscript.

### Conflict of interest statement

The authors declare that the research was conducted in the absence of any commercial or financial relationships that could be construed as a potential conflict of interest.
