# Supplementary material for: An RNA-Seq Analysis of Grape Plantlets Grown in vitro Reveals Different Responses to Blue, Green, Red LED Light, and White Fluorescent Light
Source: Front Plant Sci. 2017 Jan 31;8:78. doi: 10.3389/fpls.2017.00078 (PMC5281588; doi:10.3389/fpls.2017.00078)
Supplement: Supplementary file 1 [file DataSheet1.docx]

Supplement 1

An RNA-Seq Analysis of Grape Plantlets Grown in vitro Reveals Different Responses to Blue, Green, Red LED Light, and White Fluorescent Light

Chun-Xia Li ^1^, Zhi-Gang Xu ^1^**, Rui-Qi Dong ^2^, Sheng-Xin Chang ^3^, Lian-Zhen Wang ^1,4^, Muhammad Khalil-Ur-Rehman ^2^ and Jian-Min Tao ^2^*

*** Correspondence:
***Corresponding author: Prof. Tao Jian-Min
taojianmin@njau.edu.cn
******Co-corresponding author: Prof. Xu Zhi-Gang
xuzhigang@njau.edu.cn

# Supplement Figures and Tables

## Supplement Tables

**Table 1.** Summary of mapping result (mapping to reference genes)

| Sample ID | Total Reads | Total BasePairs | Total Mapped Reads | Perfect Match | <=2bp Mismatch | Unique Match | Multi-position Match | Total Unmapped Reads |
| --- | --- | --- | --- | --- | --- | --- | --- | --- |
| B1 | 12,038,255  (100.00%) | 589,874,495  (100.00%) | 9,004,784  (74.80%) | 6,683,781  (55.52%) | 2,321,003  (19.28%) | 8,761,357  (72.78%) | 243,427  (2.02%) | 3,033,471  (25.20%) |
| B2 | 11,850,001  (100.00%) | 580,650,049  (100.00%) | 9,025,842  (76.17%) | 6,303,963  (53.20%) | 2,721,879  (22.97%) | 8,783,644  (74.12%) | 242,198  (2.04%) | 2,824,159  (23.83%) |
| G1 | 11,717,708  (100.00%) | 574,167,692  (100.00%) | 8,072,795  (68.89%) | 6,311,550  (53.86%) | 1,761,245  (15.03%) | 7,808,740  (66.64%) | 264,055  (2.25%) | 3,644,913  (31.11%) |
| G2 | 11,567,416  (100.00%) | 566,803,384  (100.00%) | 8,724,827  (75.43%) | 6,109,340  (52.82%) | 2,615,487  (22.61%) | 8,441,112  (72.97%) | 283,715  (2.45%) | 2,842,589  (24.57%) |
| R1 | 11,871,516  (100.00%) | 581,704,284  (100.00%) | 8,105,161  (68.27%) | 6,334,462  (53.36%) | 1,770,699  (14.92%) | 7,848,858  (66.12%) | 256,303  (2.16%) | 3,766,355  (31.73%) |
| R2 | 11,584,228  (100.00%) | 567,627,172  (100.00%) | 8,716,313  (75.24%) | 6,112,368  (52.76%) | 2,603,945  (22.48%) | 8,399,808  (72.51%) | 316,505  (2.73%) | 2,867,915  (24.76%) |
| W1 | 11,965,066  (100.00%) | 586,288,234  (100.00%) | 8,746,891  (73.10%) | 6,451,403  (53.92%) | 2,295,488  (19.18%) | 8,478,120  (70.86%) | 268,771  (2.25%) | 3,218,175  (26.90%) |
| W2 | 11,450,416  (100.00%) | 561,070,384  (100.00%) | 8,623,429  (75.31%) | 6,013,935  (52.52%) | 2,609,494  (22.79%) | 8,360,478  (73.01%) | 262,951  (2.30%) | 2,826,987  (24.69%) |

**Table 2.** Summary of mapping result (mapping to reference genome)

| Sample ID | Total Reads | Total BasePairs | Total Mapped Reads | Perfect Match | <=3bp Mismatch | Unique Match | Multi-position Match | Total Unmapped Reads |
| --- | --- | --- | --- | --- | --- | --- | --- | --- |
| B1 | 12,038,255  (100.00%) | 589,874,495  (100.00%) | 10,018,796  (83.22%) | 7,127,408  (59.21%) | 2,891,388  (24.02%) | 9,545,018  (79.29%) | 473,778  (3.94%) | 2,019,459  (16.78%) |
| B2 | 11,850,001  (100.00%) | 580,650,049  (100.00%) | 9,956,240  (84.02%) | 6,706,176  (56.59%) | 3,250,064  (27.43%) | 9,503,300  (80.20%) | 452,940  (3.82%) | 1,893,761  (15.98%) |
| G1 | 11,717,708  (100.00%) | 574,167,692  (100.00%) | 9,755,858  (83.26%) | 7,230,460  (61.71%) | 2,525,398  (21.55%) | 9,149,138  (78.08%) | 606,720  (5.18%) | 1,961,850  (16.74%) |
| G2 | 11,567,416  (100.00%) | 566,803,384  (100.00%) | 9,713,257  (83.97%) | 6,551,518  (56.64%) | 3,161,739  (27.33%) | 9,182,918  (79.39%) | 530,339  (4.58%) | 1,854,159  (16.03%) |
| R1 | 11,871,516  (100.00%) | 581,704,284  (100.00%) | 9,855,449  (83.02%) | 7,297,911  (61.47%) | 2,557,538  (21.54%) | 9,243,292  (77.86%) | 612,157  (5.16%) | 2,016,067  (16.98%) |
| R2 | 11,584,228  (100.00%) | 567,627,172  (100.00%) | 9,723,373  (83.94%) | 6,596,023  (56.94%) | 3,127,350  (27.00%) | 9,177,786  (79.23%) | 545,587  (4.71%) | 1,860,855  (16.06%) |
| W1 | 11,965,066  (100.00%) | 586,288,234  (100.00%) | 9,941,763  (83.09%) | 7,034,871  (58.80%) | 2,906,892  (24.29%) | 9,412,970  (78.67%) | 528,793  (4.42%) | 2,023,303  (16.91%) |
| W2 | 11,450,416  (100.00%) | 561,070,384  (100.00%) | 9,639,220  (84.18%) | 6,489,486  (56.67%) | 3,149,734  (27.51%) | 9,161,518  (80.01%) | 477,702  (4.17%) | 1,811,196  (15.82%) |

## Supplement Figures


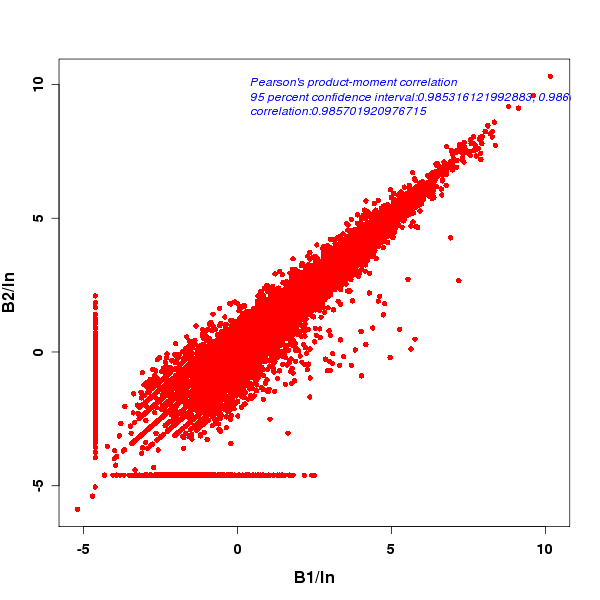

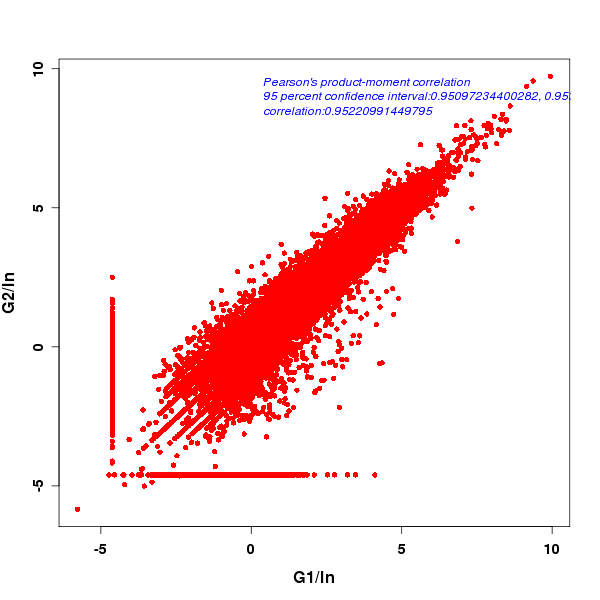

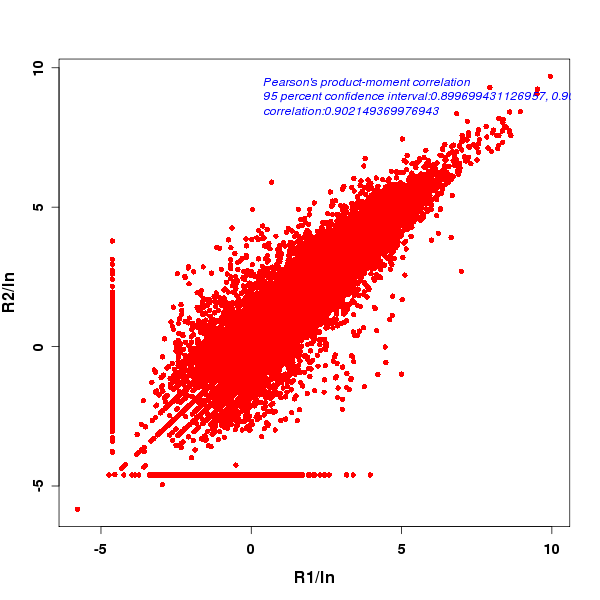

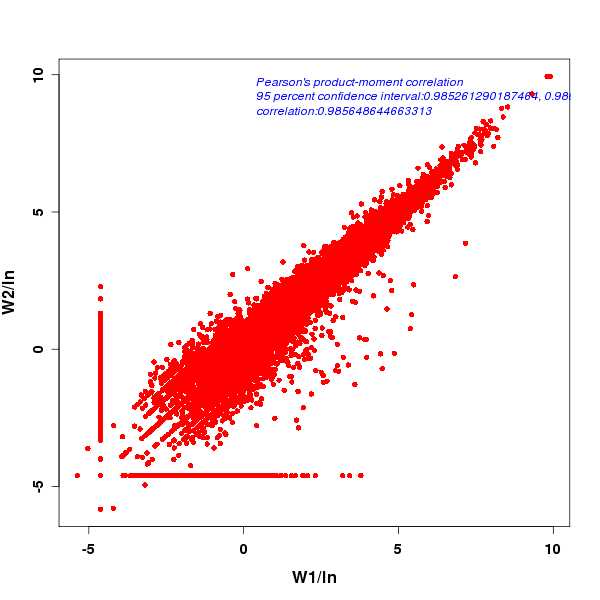


**Figure 1.** Correlation analysis among same group.


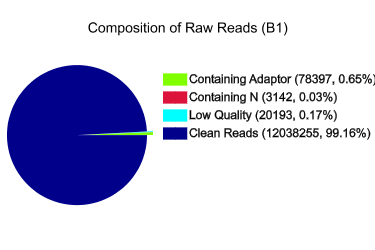

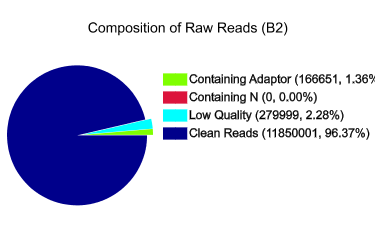

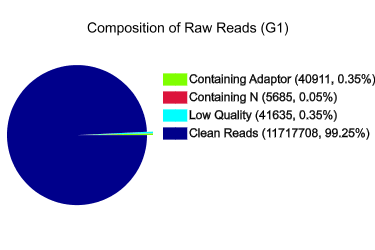

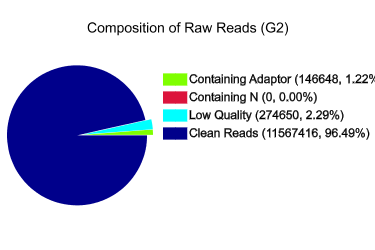

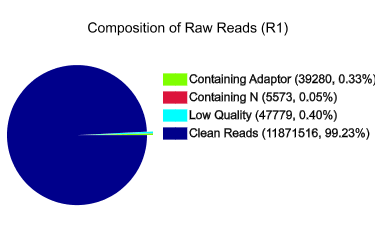

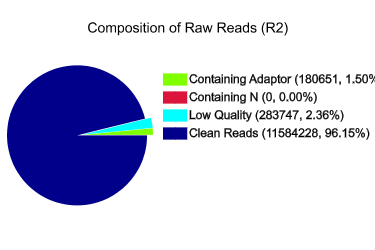

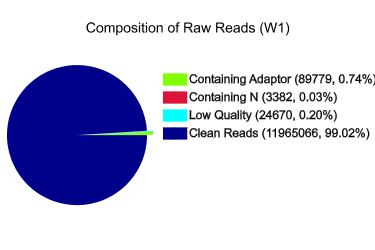

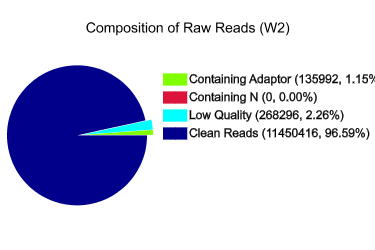


**Figure 2.** Distribution of reads from four samples group of leaf of grape plantlets in vitro.


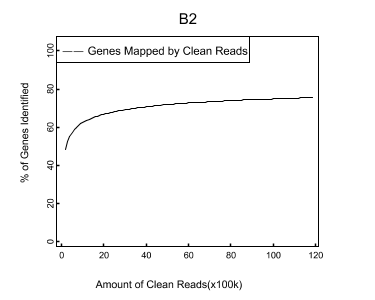

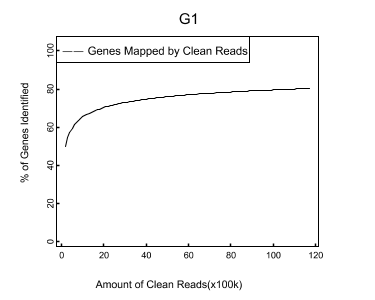

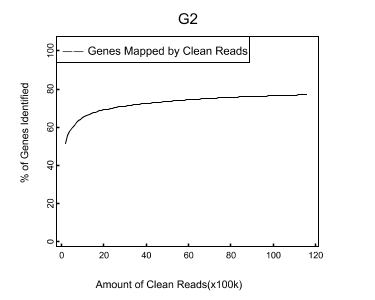

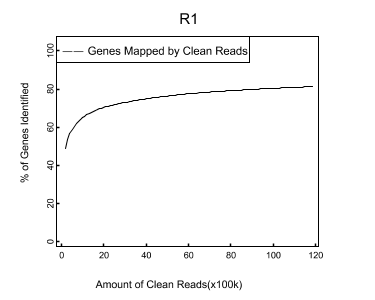

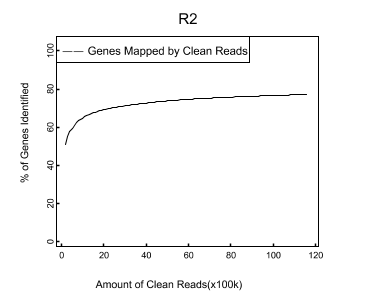

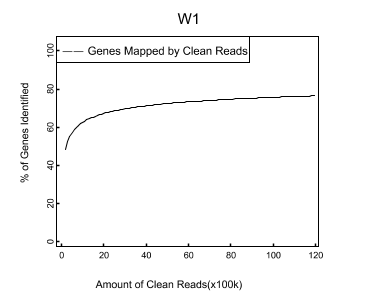

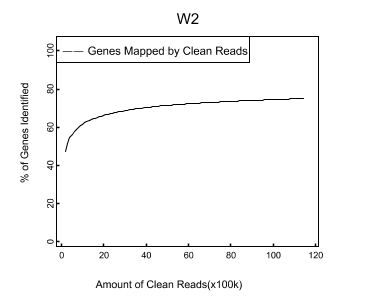

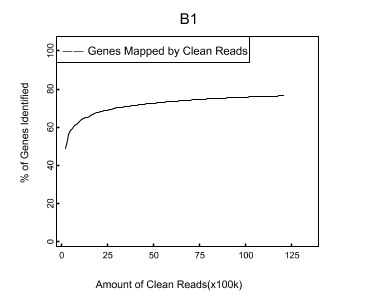


**Figure 3.** Image of saturation analysis of reads number and mapped genes.


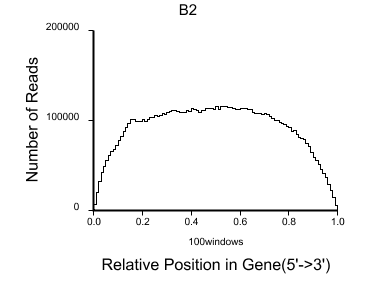

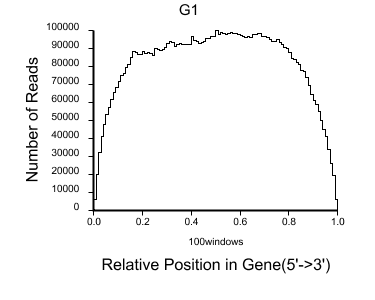

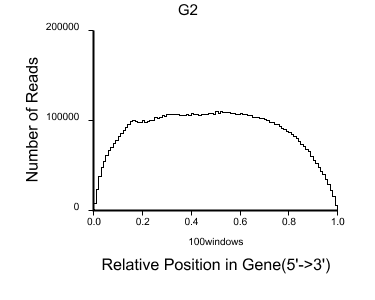

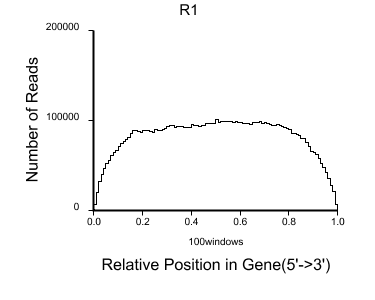

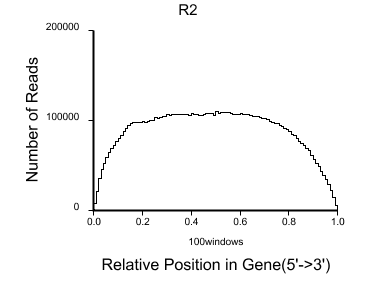

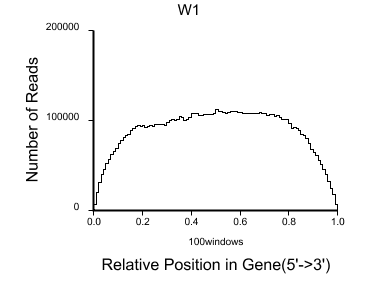

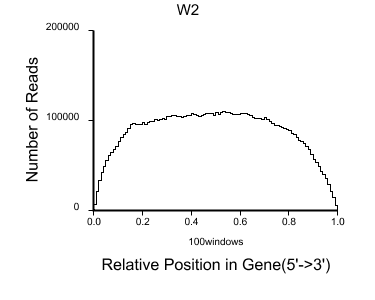

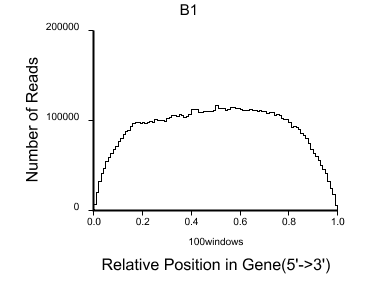


**Figure 4.** Distribution statistics of reads mapped to reference gene.


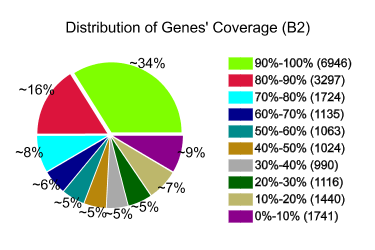

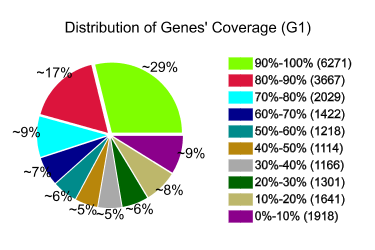

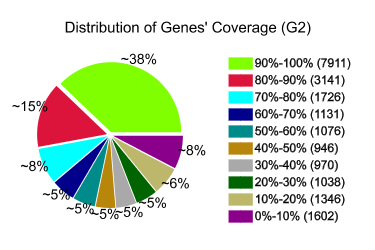

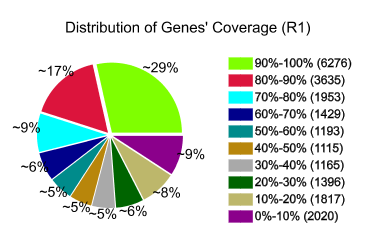

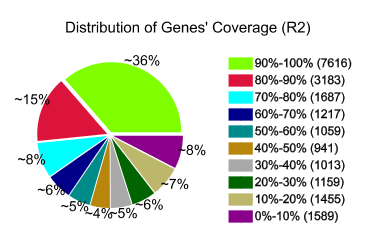

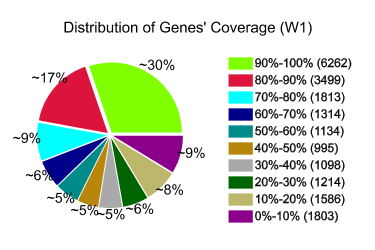

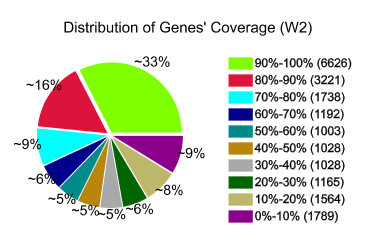

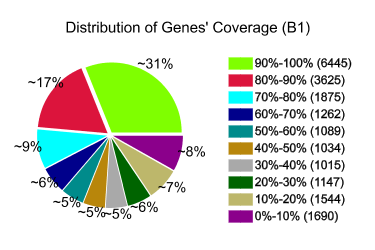


**Figure 5.** Image of statistics of gene coverage

**Figure 6.** Statistic chart of DEGs in different treatment groups of light-emitting diode light source.


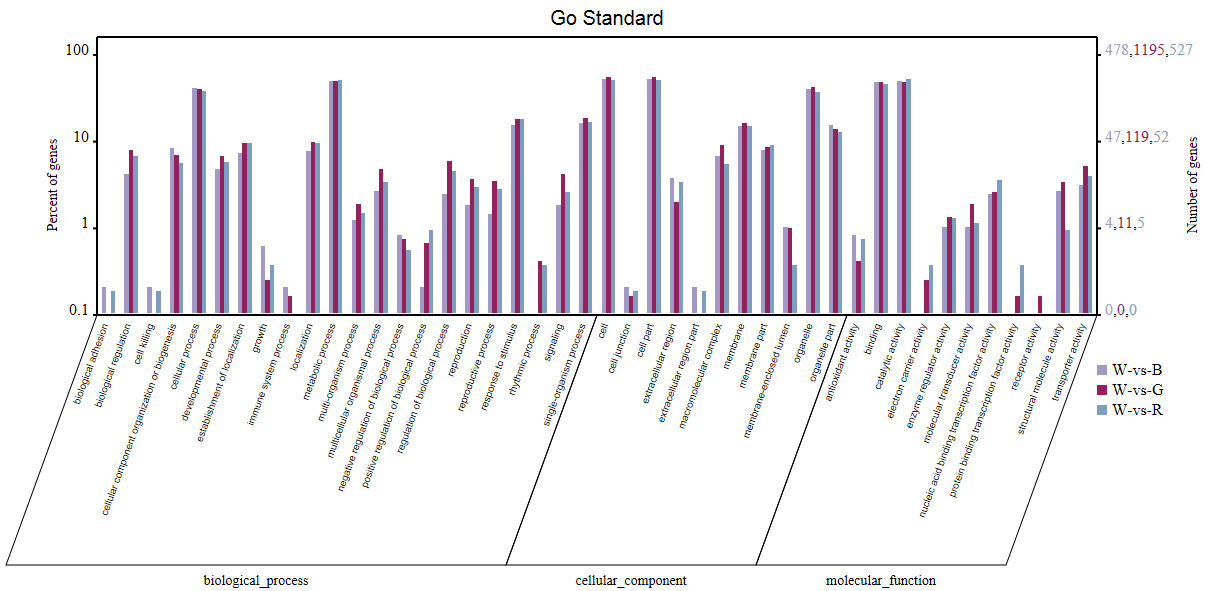


**Figure 7.** GO classification of DEGs.


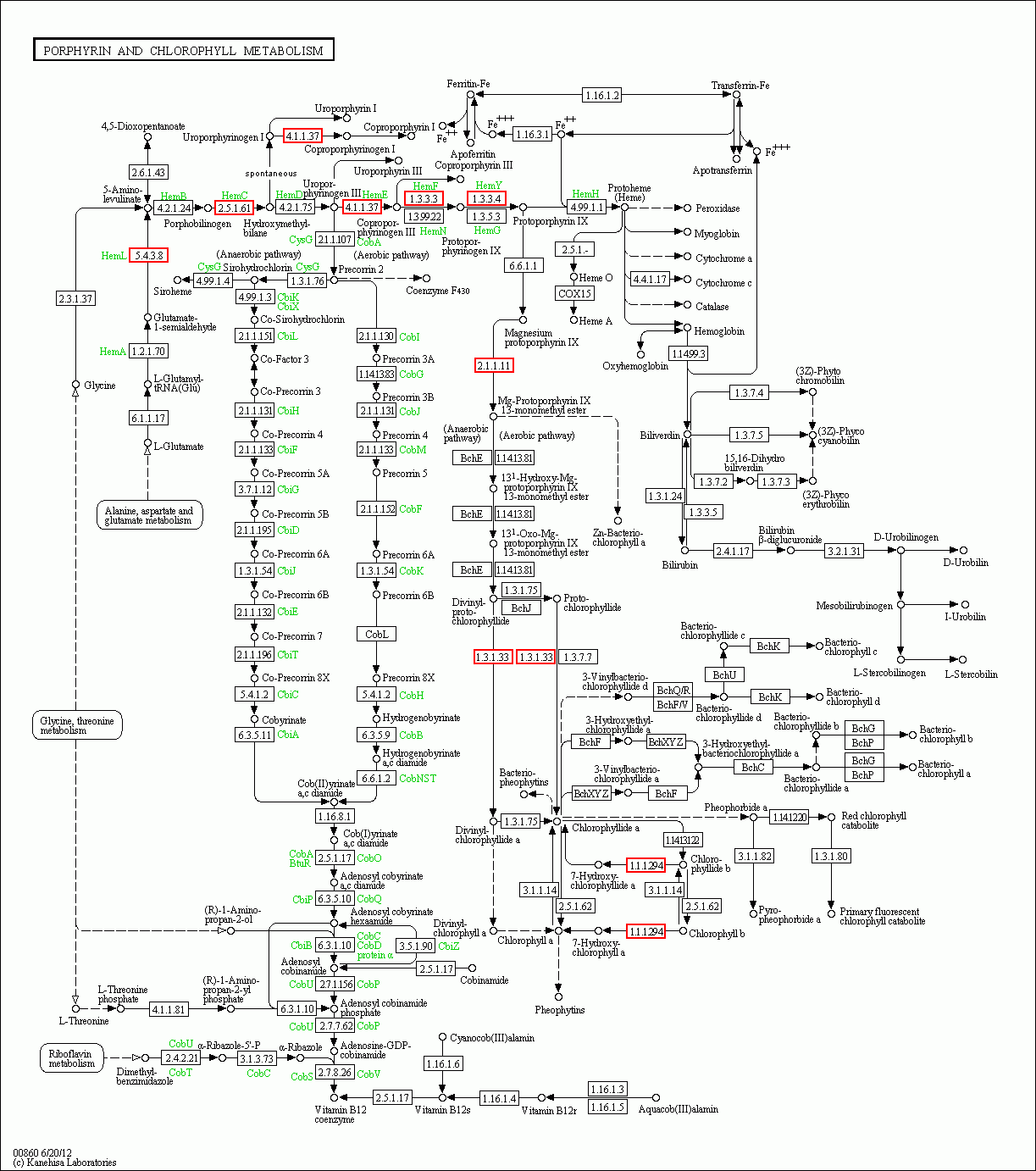


**Figure 8.** Porphyrin and chlorophyll metabolism of blue light treatment compared to the white light treatment. The red box denotes up-regulated expression of genes for the blue light treatments compared to the white light treatment.
